# Random Forest Regressor-Based Approach for Detecting Fault Location and Duration in Power Systems

**DOI:** 10.3390/s22020458

**Published:** 2022-01-08

**Authors:** Zakaria El Mrabet, Niroop Sugunaraj, Prakash Ranganathan, Shrirang Abhyankar

**Affiliations:** 1 School of Electrical and Computer Science, University of North Dakota, Grand Forks, ND 58202, USA; niroop.sugunaraj@und.edu (N.S.); prakash.ranganathan@und.edu (P.R.); 2 Electricity Infrastructure and Buildings Division, Pacific Northwest National Laboratory, Richland, WA 99354, USA; shrirang.abhyankar@pnnl.gov

**Keywords:** three-phase fault, random forest regressor, missing and streaming data, GridPACK

## Abstract

Power system failures or outages due to short-circuits or “faults” can result in long service interruptions leading to significant socio-economic consequences. It is critical for electrical utilities to quickly ascertain fault characteristics, including location, type, and duration, to reduce the service time of an outage. Existing fault detection mechanisms (relays and digital fault recorders) are slow to communicate the fault characteristics upstream to the substations and control centers for action to be taken quickly. Fortunately, due to availability of high-resolution phasor measurement units (PMUs), more event-driven solutions can be captured in real time. In this paper, we propose a data-driven approach for determining fault characteristics using samples of fault trajectories. A random forest regressor (RFR)-based model is used to detect real-time fault location and its duration simultaneously. This model is based on combining multiple uncorrelated trees with state-of-the-art boosting and aggregating techniques in order to obtain robust generalizations and greater accuracy without overfitting or underfitting. Four cases were studied to evaluate the performance of RFR: 1. Detecting fault location (case 1), 2. Predicting fault duration (case 2), 3. Handling missing data (case 3), and 4. Identifying fault location and length in a real-time streaming environment (case 4). A comparative analysis was conducted between the RFR algorithm and state-of-the-art models, including deep neural network, Hoeffding tree, neural network, support vector machine, decision tree, naive Bayesian, and K-nearest neighborhood. Experiments revealed that RFR consistently outperformed the other models in detection accuracy, prediction error, and processing time.

## 1. Introduction

Fault identification is critical for seamless power grid operation. Utilities are working around the clock to reduce outage rates from interruptions such as contact with natural vegetation, animals, or weather events [1,2,3]. The unplanned outages can lead to long service interruptions and significant economic impact to the customers. The cost to various consumers for a one-hour outage during a summer afternoon was estimated to be approximately USD 3 for a typical customer, USD 1200 for small and medium organizations, and USD 82,000 for large organizations [4]. These outage costs increased substantially depending on the time of year and outage duration, especially when they occur during winter. Thus, predicting faults in the system along with their duration is the first step towards reducing the number of unplanned outages and providing a prediction-based plan to the utility for deploying the appropriate maintenance crews and the sequence of operations [5,6].

Physical faults in power systems are generally classified as balanced or unbalanced faults [7,8]. An unbalanced fault, or asymmetrical fault, is a commonly occurring fault that can be series or shunt type. The voltage and frequency values increase and the current level decreases during a fault phase in the series fault type. The current level rises and the frequency and voltage levels decrease during a fault phase in the shunt fault type. There are several shunt fault types: single line to ground (SLG), line to line (LL), double line to ground (DLG), and three-phase to the ground (LLL). An SLG occurs when a transmission line phase touches a neutral wire or ground. The DLG or LL faults occur when two or more phases make a connection with the ground, primarily due to natural weather events such as storms, high winds, or fallen trees. The cause may also be due to equipment failure, line connecting the remaining phases, or a failing tower. The likelihood of occurrences for each fault type is 70% for SLG, 15% for LL, 10% for DL, and 5% for LLL [8,9]. An LL fault occurrence is rare; however, it is categorized as a severe fault that can increase fault current magnitude, resulting in outages or severe grid asset damage. These damages underscore the need for a fault detection and location identification model.

Many power system fault detection approaches have been reported in peer-reviewed literature. The authors in [7] provide a review of conventional and machine-learning-based techniques for fault location identification. Some conventional approaches include the traveling wave model, the impedance-based method, and synchronized voltage and current-based measurements. The traveling wave approach requires high-speed data acquisition equipment, a Global Positioning System (GPS), sensors, and transient fault recorders to detect the transient waveform. The location of the fault is computed by “time-tagging the arrival of the traveling wave at each end of the line and comparing against the time difference to the total propagation time of the line with the help of GPS” [10]. This approach has several advantages, as it is not impacted by excessive resistance, load variance, reflection, grounding resistance, traveling wave refraction, or series capacitor bank [7]; however, the accuracy of the approach relies on capacitance and line inductance. The impedance-based approaches [8,11] are simple and easy to implement, unlike the time-wave method, as they only require measurement data that include fault voltages and fault currents collected from the digital fault recorder or relays. The accuracy of this approach can be affected in the circumstances such as a grounded fault, where the resistances can reach higher values.

Machine learning (ML)-based approaches for detecting fault locations have been reported in peer-reviewed literature. In these approaches, training data was generated using inputs such as voltage, current, and phase angle, and using fault location as an output. The authors in [12] proposed a back propagation-based neural network (BPN) to estimate fault location in distribution networks. The fault current is a critical feature for training the NN model. A Levenberg–Marquardt algorithm, also known as damped least square, was applied to BPN for faster convergence. The BNN model was then deployed to run on the DIgSILENT Power Factory 13.2. A feed-forward NN (FNN) based approach was proposed in [13], where fault voltages and fault currents were selected as the two features to train the model. A sigmoid activation function was used to normalize the data. The results indicated a detection error of less than 3%. Another NN-based approach was proposed in [14], to estimate fault distances from substations. The selected input features included three-phase voltage, current, fault conditions, and active power gathered from the substations. This approach was trained on different fault locations, resistances, and loads, then tested on an IEEE 34-bus system. This method yielded promising results, even with dynamic changes in network topology and higher noise tolerance. The authors of [15] proposed a convolutional neural network (CNN)-based approach using bus voltages. This method was trained and tested on IEEE 39-bus and IEEE 68-bus systems under uncertain conditions for system observability and measurement quality. Their results indicated that CNN can localize a faulted line in low-visibility conditions in 7% of the buses. High accuracy was reported for NN-based approaches in the studies mentioned above; however, the training time required for NN was longer and was not suitable for dynamic or real-time environments. In [16], authors discussed a fault line identification and localization approach using random forest (RF) and decision tree classifiers. The obtained experiment results show a classification accuracy of 91%. Another RF-based approach was proposed in [17]. Here, the model was trained on three-phase current and voltage data, validated using a IEEE-34 system, and achieved an accuracy above 90%.

A fault-detecting KNN-based approach in a photovoltaic (PV) system was proposed in [18]. This approach was trained and tested on data generated from a developed PV model. The reported results indicated a classification accuracy of 98.70%, with error values ranging between 0.61% and 6.5%. The authors in [19] proposed a real-time event classification and fault localization approach for a synchrophasor dataset. Their methodology relied on three processes: 1. Removing bad data from collected PMU measurements using the maximum likelihood estimation (MLE) approach; 2. Events were classified using a combination of density-based spatial clustering and applications with noise (DBSCAN). Logic rules were generated using a physics-based decision tree (PDT) method that uses parameters such as active power, reactive power, and fault event types; and 3. Reporting localized events in real time using graph-theory. Three case studies were analyzed using metrics such as precision and recall. A score metric was computed using Shannon entropy and descriptive statistical parameters, such as standard deviation, range, mean difference, and crest factor. The authors determined that their proposed data cleansing approach outperformed Chebyshev and K-means methods, with 95% precision. The average classification run-time algorithm was approximately 0.09 s for a typical window size of 30 samples involving PMU sensors.

A hybrid method using wavelet transform and support vector machine (SVM) was examined in [20] to locate faults in transmission lines, with the methods described in two stages. Voltage and current values emitted by a transmitter were used to locate the fault in the first phase. The second phase fed a multi-class SVM model for training based on selected influential features, with the classification of fault locations completed using a regression approach. The fault classification error was below 1% for all fault types: 0.26% for SLG, 0.74% for LLG, 0.20% for LL, and 0.39% for LLLG. This approach was faster and relatively accurate, even for larger-sized datasets; however, it requires the careful selection of appropriate kernel type and hyperparameters. The authors of [21] proposed an event location estimation (ELE) algorithm for the wide-area PMU data monitoring system. Their approach relied on clustering and wavelet analysis to detect and localize events in real time. The network was initially divided into several clusters, where each cluster was defined as an electrical zone (EZ) using K-means. A wavelet-based event approach was used to detect and localize event occurrences by tracking any significant disturbance levels, such as event magnitudes. Once the event was detected, its magnitude was defined using a modified wavelet energy (MWE) value, with locations estimated at each EZ. The authors implemented the ELE approach in a real-world PMU-setting containing 32 dynamic events with exceptional localizing accuracy values. They did not consider data quality issues in the PMU measurements; therefore, some error may have been introduced by irregular sampling, data rate, bandwidth challenges, and time synchronization errors.

The authors of [22] discussed a wavelet decomposition technique combined with fuzzy logic to identify fault lines and locations in a multi-terminal high-voltage direct current (MTHVDC) network. The wavelet coefficients of positive and negative currents were initially computed and then fed to a fuzzy logic-based voting system to identify the fault lines. Once the line was identified, a traveling wave-based algorithm was used to determine the exact fault location using the Daubechies wavelets. A discrete wavelet transform (DWT) combined with SVM for distribution network fault detection was proposed in [23]. The features were extracted using SVM and decision trees (DT), then optimized using a genetic algorithm (GA). The author’s model performance was evaluated on two active distribution networks, IEEE 13-bus and IEEE 34-bus systems. They reported that their model outperforms the probabilistic neural network (PNN).

Compared with detecting fault location, relatively few works have been carried out to predict fault duration. However, this is arguably pertinent information from the customer’s perspective. When a fault or an outage occurs and consumers ask when the power will be restored, utilities have to provide an accurate estimation of the recovery time. Seattle City Light provides a real-time outage map with an estimated restoration time; however, the difference between the actual outage duration and the estimation time is large, possibly because of the conventional techniques used [5,24]. There are few approaches in the literature that have attempted to address this issue. Authors in [5] proposed a real-time approach for detecting outages in distributed systems. This approach was based on recurrent neural network (RNN) and was trained on three sources of historical data: outage report provided by Seattle City Light and 15 years of data, repair logs, and weather information. Another approach for predicting faults duration in transmission system was proposed in [6]. This approach was based on naive Bayes classifier (NBC) and support vector machine (SVM) and it was trained on nontemporary fault-type data including features such as substation, asset type, fault category, and outage start time. The reported results indicated an accuracy above 97%.

Table 1 provides a summary of the relevant fault detection approaches along with their advantages and potential limitations. We propose a random forest regressor (RFR)-based model to detect fault locations and predict their duration simultaneously. From a machine learning perspective, fault location detection is usually approached as a multiclass classification problem where the output would be a class label, fault position; while fault duration prediction is regarded as a regression problem as the output would be a continuous value, fault duration. This novel work addresses these two issues using a single approach. By mapping faults location and duration output values into one single output; these two problems can be concurrently addressed through a single regression model. The proposed model is novel due to the following reasons. 1. It detects fault locations and predicts their duration simultaneously; 2. It is adaptable to other case scenarios and power system datasets as it includes an ensemble of multiple uncorrelated trees that achieves strong generalizations; 3. It predicts various fault duration including short, medium, and large duration; 4. It is convenient for real-time applications as it requires less processing time compared to the existing approaches. GridPACK framework [25] was used to train the model by simulating several three-phase fault scenarios on a nine-bus system to generate appropriate datasets. A collection of four experiments are formulated to evaluate the performance of the RFR model. The model was evaluated in experiment 1 for fault detection accuracy, then compared to seven classifiers: neural network (NN), deep neural network (DNN), support vector machine (SVM), k-nearest neighbors (KNN), naive Bayes (NB), decision tree (DT), and Hoeffding tree (HT). The RFR model was evaluated in experiment 2 for predicting fault duration, then compared to the regression version of models such as support vector regressor and decision tree regressor. Mean squared error (MSE) and mean absolute error (MAE) were used as evaluation metrics. In experiment 3, the RFR was examined in terms of handling missing data possibly caused by equipment failure, data storage issues, or unreliable communication. The RFR was tested in a streaming data environment in experiment 4, where multiple window sizes were considered. The MSE and processing time for the RFR were then compared to HT and DNN. The HT and DNN models are commonly suggested for power system streaming data [26,27,28].

This paper is organized into the following sections: Section 2 focuses on RFR model description, with details on simulated fault scenarios, feature selections, and training/testing process; Section 3 discusses the analysis of four experiment scenarios for classifying and predicting fault location and duration with off-line/streaming conditions; and Section 4 draws conclusions and recommendations for future work.

## 2. Methodology

### 2.1. Random Forest Regressor (RFR) Model

Random forest *F* is an ensemble approach with several independent and uncorrelated decision trees $F=\{t_{1},t_{2},\cdots ,t_{t}\}$. These uncorrelated trees assist model *F* in achieving an accurate generalization by injecting randomness into the decision trees [31]. These generalizations rely on the application of a bagging technique, which combines the concepts of bootstrapping and aggregation [31]. Consider a training set $S={\left\{X^{m},Y^{m}\right\}}_{\left(M=1\right)}^{m}$, where $X\subset R^{D}$ and consists of input feature space with parameters such as voltage (v), phase angle (ϕ), current, and frequency (*f*). *Y* is a multidimensional continuous space $Y\subset R^{D^{\prime }}$, and includes both the fault location and corresponding fault duration. *M* is the number of samples, and bootstrap is a subset $S_{t}$ of the entire training set *S*, where each instance has been randomly sampled using a uniform distribution with or without replacement. The resulting bootstrap data includes the same number of instances as the original data set *S*; however, approximately 1/3 of these samples are duplicated and approximately 1/3 of the instances are removed from the bootstrap sample. Multiple passes are performed on the input data to create bootstraps for each tree. Once the training and testing is completed on the bootstrap data, the prediction of all the independent trees are averaged as one aggregated value.

Assuming that output variables follow a multivariate Gaussian distribution with mean μ and covariance Σ, the regression posterior can be modeled as

(1) $$P\left(y|x,P_{t}\right)=N_{t}\left(y|\mu_{t},\Sigma_{t}\right)$$

where $P_{t}$ is a partition built by a random tree $t_{t}$, $N_{t}$ is a multivariate Gaussian with mean $\mu_{t}$, and covariance $\Sigma_{t}$ is predicted in the output space Y from the subsets of the training dataset. The purpose of training the trees is to reduce the uncertainty related to the multivariate Gaussian model, especially when an appropriate splitting function f must be selected to split the subset $S_{l}$ of the training set. These calculations are performed at each arriving node $N_{l}$ in the tree $t_{t}$ to reduce any prediction uncertainty caused due to “splitting”.

An example of function *f* includes information gain and the Gini index. The unweighted differential entropy function, which is a continuous version of Shannon’s entropy (SE), is considered an optimal function for computing information gain in a regression task [32,33]. The SE function was selected, as it reported satisfactory results in terms of prediction error, defined as

(2) $$f\left(S_{l}\right)=\int_{\left(y\in Y\right)}\sum_{i=1}^{n}P\left(y|S_{l}\right)log\left(P\left(y|S_{l}\right)\right)dY$$

where *i* is a given input instance and *y* is an output including both fault duration and location.As we model the posterior using multivariate Gaussian, *f* can be rewritten as [34]

(3) $$f\left(S_{1}\right)=\frac{1}{2}\log\left({\left(\pi \exp\right)}^{D\prime }|\Sigma^{\left(S_{l}\right)}|\right)$$

where $\Sigma^{\left(S_{l}\right)}$ is the covariance matrix estimated from the subset $S_{l}$. After splitting the subset $S_{l}$ at node $N_{l}$ into two subsets nodes, $S_{l}^{right}$ and $S_{l}^{left}$, using function *f*, the information gain Δ is calculated using

(4) $$\Delta =f\left(S_{l}\right)-w_{l}f\left(S_{l}^{left}\right)-w_{r}f\left(S_{l}^{right}\right)$$

where $w_{l}=\frac{|S_{l}|}{|S_{l}^{left}|}$ and $w_{r}=\frac{|S_{l}|}{|S_{l}^{right}|}$. Once the training phase is completed, the prediction phase consists of sending the new received instances through the trees of the forest and the posteriors of all the trees are estimated using the following equation:

(5) $$P\left(y|x\right)=\frac{1}{T}\sum_{t=1}^{T}P\left(y|x,P_{t}\right)$$

where *T* is the number of trees in the forest and $P_{t}$ is the partition introduced by $t_{t}$. Given any new instance, the model can predict its corresponding fault duration and location by maximizing a posterior:

(6) $$\hat{Y}=argmax_{y\in Y}P\left(y|x\right)$$

### 2.2. Dataset

**Figure 1:**
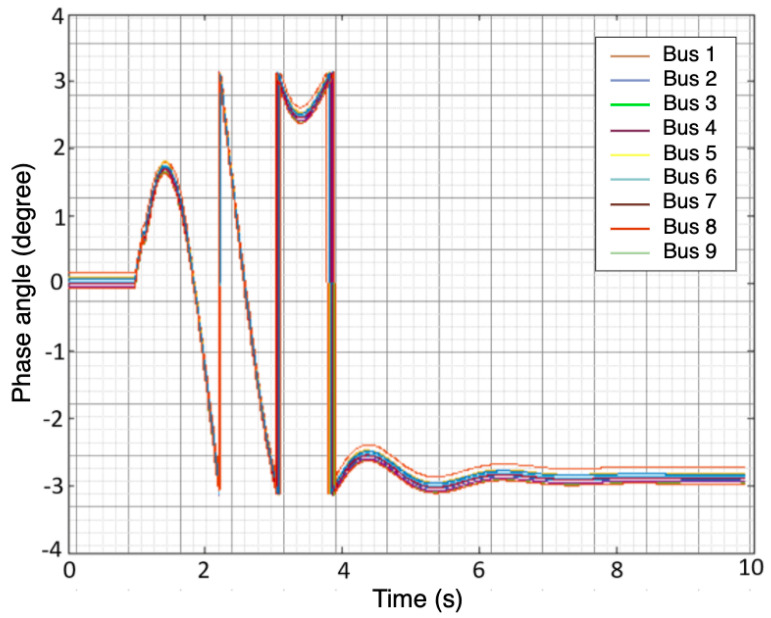
Phase angle of the 9 buses after injecting three—phase fault.

**Figure 2:**
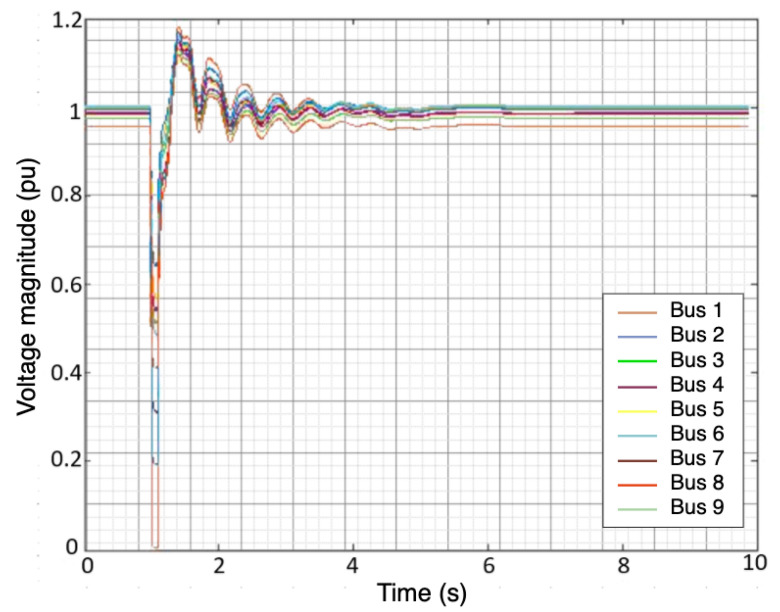
Voltage magnitude of the 9 buses after injecting three—phase fault.

**Figure 3:**
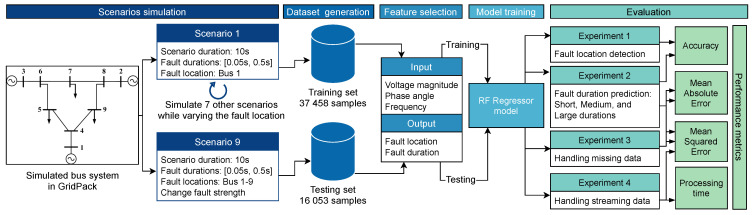
Conceptual diagram of the proposed approach.

The simulated fault scenarios were completed using GridPACK software, an open-source framework designed to support the development and implementation of power grid applications. Examples of these applications include power flow simulations for the electric grid, contingency analysis of the power grid, state estimation based on electric grid measurements, and the dynamic simulation of the power grid. These applications are capable of running on high-performance computing architecture (HPC) [25]. The dynamic simulation application package in GridPACK was selected to simulate a three-phase fault at various bus locations with different fault duration(s) using a nine-bus system. The faults duration were varied from 0.05 to 0.5 s along with fault strength levels, such as magnitude. An example of scenario one is depicted in Figure 1 and Figure 2. Three features were selected to capture both the fault location and duration: the voltage magnitude ($V_{m}$) at each bus, the phase angle (ϕ) at each bus, and the frequency (*f*) of the generators. The timing of the fault applied to each bus was ten seconds. The total number of samples for all simulated scenarios equaled 53,512 samples. A summary of the training and test data is listed in Table 2. Additionally, Figure 3 illustrates a conceptual diagram of the proposed approach starting from simulation fault scenarios to evaluating the RFR model’s performances.

## 3. Results and Discussion

### 3.1. Experiments and Metrics

Four experimental scenarios were considered for the evaluation of the RFR model performance. The proposed model was assessed based on the accuracy metric in experiment 1, which is the ratio of the correctly classified fault location cases over the total number of cases. The accuracy metric can be expressed as

(7) $$Accuracy=\frac{\left(TP+TN\right)}{\left(TP+TN+FP+FN\right)}$$

where TP is the true positive, TN is the true negative, FP is the false positive, and FN is the false negative. These values were obtained from the confusion matrix. The second set of experiments evaluated the model’s performance when predicting the fault duration. As this feature is a continuous value, the accuracy metrics cannot be used; therefore, other performance metrics, such as MAE and MSE, were selected. The MAE is the average of the absolute differences between the actual and predicted fault duration, and it is given by

(8) $$MAE=\frac{1}{n}\sum_{i=1}^{n}|\;\left(\hat{y}-y\right)\;|$$

where $\hat{y}$ is the predicted fault duration, *y* is the actual fault duration, and *n* is the number of instances or cases. Unlike MAE, MSE has the benefit of penalizing for significant errors because it averages the squared differences between the actual fault duration and the predicted one, expressed as

(9) $$MSE=\frac{1}{n}\sum_{i=1}^{n}{\left(\hat{y}-y\right)}^{2}$$

The MSE and MAE of the proposed model were compared to the regression version of the other seven models listed above. The fault duration and location were evaluated in a streaming window environment during experiment 3.

### 3.2. Models Hyperparameters Tuning

To conduct a fair comparison between RFR and the other models, a hyperparameter study was conducted to determine the optimal parameters. Two approaches can be investigated to select the best hyperparameters: GridSearch and RandomizedSearch [35]. The former is convenient for an exhaustive search for the best-performing hyperparameters given advanced computing resources, whereas the latter defines a grid of hyperparameters and randomly selects the optimal one [35]. GridSearch was employed to examine, in depth, the relevant parameters for each model and their optimal values using a subset of the data. For KNN, two weighting functions were chosen with varying numbers of neighbors: uniform and distance. In uniform weighting, all points within the neighborhood are weighted equally, while in distance weighting, closer neighbors are given more weight [36]. In the RFR method, two maximum features methods were selected, sqrt and log2, to determine the number of features to consider when looking for the best split. For SVM, two kernel types were chosen, polynomial and radial basis function (RBF). A range of regularization parameters (C) was also considered. For NN, two activation functions were selected in conjunction with a variety of hidden nodes. In DT, the minimum number of samples needed to split a node internally was determined; additionally, various values were investigated to control randomness within the tree. Alpha and lambda were selected as the shape parameters for NB; alpha is the shape parameter for the Gamma distribution prior to alpha, and lambda is the shape parameter for the Gamma distribution prior to lambda [36]. For DNN, two numbers of hidden layers were chosen, each of which has multiple hidden neuron nodes. Finally, two split functions were investigated for HT with varying split confidence values.

Table 3 provides the GridSearch methods results for the various models. The optimal parameters for each model are highlighted. KNN reported the lowest MSE values, 6.7 and 0.16 standard deviation, with the distance weight function and 100 neighbors. According to these results, KNN fits data more smoothly with increasing number of neighbors; this is due to the fact that more neighbors reduces the edginess by taking into account more data, thus lowering the overall error of the model. SVM reaches low error, 5.9, when using RBF kernel and a regularization parameter (C) set to 10. These results reflect that increasing the C value can contribute to low error rates, possibly because there are more potential data points within the margin or that were incorrectly classified, which can be corrected by using a high C value. DT performs better with a leaf size of six and a random state of one. Based upon these results, it appears that increasing the minimum leaf size will increase the model’s ability to determine the appropriate pruning strategy, and, as a result, improve its performance.

The optimal DNN configuration entails five hidden layers, each of which contains 100 hidden nodes with relu function. These results suggest that the number of hidden layers and hidden neuron nodes did not dominate the model’s performance; that is, the model obtained the best results without overfitting by using five hidden layers, each containing 100 neuron nodes. The RFR showed optimal results using log2 as a maximum feature and 100 trees. When splitting a node with log2 as a maximum feature, RFR is better able to find the optimal size of the random subset of features. An optimal configuration of NN includes a relu function and 150 hidden nodes. NN results indicate that an increased number of trees does not improve the model’s performance, but rather the choice of an activation function; Relu has demonstrated a lower error rate than identity. The optimal alpha and lambda settings for NB were set to 1 × 10^−6^ and 1 × 10^−2^, respectively. NB’s results indicated that changes to alpha or lambda values do not have a significant impact on model performance. HT’s optimal parameters are information gain, as a split function, and a split confidence set to 1 × 10^−5^. HT’s results indicated that selecting a lower confidence level while using information gain reduced error rates and their standard deviations significantly.

### 3.3. Experiment Result #1: Fault Location Detection

**Figure 4:**
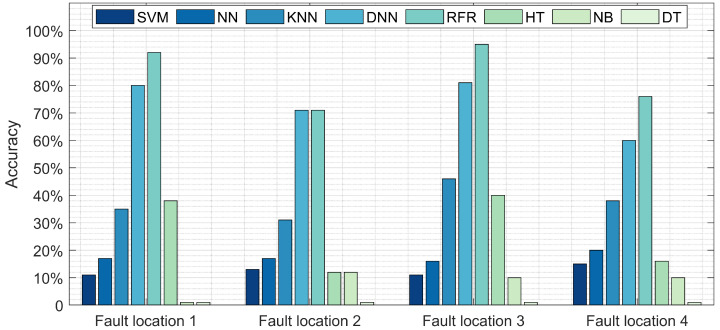
Comparison between the proposed model (RFR) and NN, DNN, SVM, NB, DT, and HT in terms of fault location detection accuracy at various location.

The results of experiment 1 are depicted in Figure 4. This figure explores the comparison between the proposed model (RFR) and the other seven models in terms of fault location detection accuracy at nine different fault locations. At fault location 1, the RFR approach detects approximately 92% of the faults, followed by DNN with 80% accuracy. NB reports the poorest performance with an accuracy rate below 2%. At the second location, DNN and RF report similar results, 71%, followed by KNN, NN, and SVM. At the third fault location, RFR reports the highest accuracy rate, 94%, followed by DNN with 78%, then KNN with 46%. RFR detects 76% of the faults at fault location 4, compared to DNN at 60%. Table 4 provides the processing time along with accuracy for the training and testing of these models. Although the testing time for the NN, NB, and DT models are relatively low, the accuracy was under 20%. Alternatively, RFR and DNN reported respective accuracy of 84% and 72% with a short test time below 0.046 s.

### 3.4. Experiment Results #2: Fault Duration Prediction

**Figure 5:**
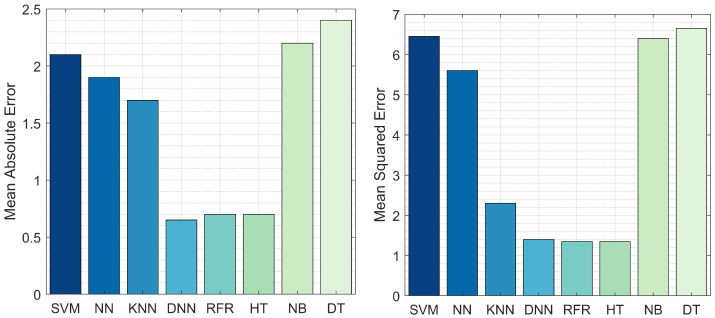
The MAE and MSE of RFR, NN, DNN, SVM, NB, DT, and HT in terms of fault duration prediction.

**Figure 6:**
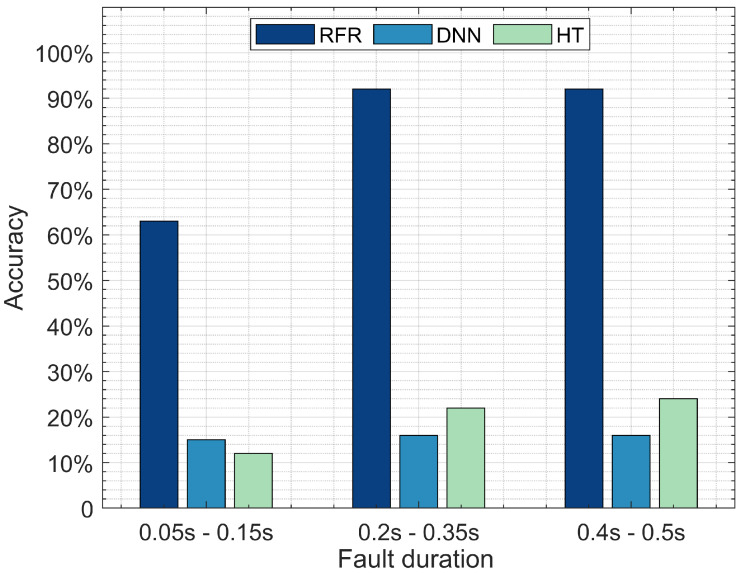
Accuracy of RF, DNN, and HT in terms of detecting fault location with various duration.

The fault duration prediction results are illustrated in Figure 5. The lowest reported MAE values were from the RFR, HT, and DNN models. The highest MAE value was from DT, at 2.4 s. These results suggest that DNN, HT, and RFR are the optimal models for predicting fault duration as the difference between the actual and predicted duration for the entire testing dataset was less than 0.6 s. Figure 6 also depicts the MSE of RFR compared to the other models. The RFR and HT models reported the lowest MSE value, close to 1 s; however, the prediction error for DNN was more than 1.5 s. The RFR, HT, and DNN models yield optimal results for MAE and MSE; therefore, these models were selected for the next experiment.

Figure 6 illustrates fault location detection by comparing the three optimal performing models, DNN, RF, and HT, tested with three different fault durations: a short fault duration ranging between 0.05 and 0.15 s, a medium fault duration ranging between 0.2 and 0.35 s, and a long fault duration ranging between 0.4 and 0.5 s. The RFR model outperforms DNN and HT when detecting faults with short, medium, and long durations. The RFR model reports a 65% accuracy when detecting the short fault duration, followed by DNN with 12%, then HT with 10%. The accuracy of the RFR model increases to 91% detection for the medium duration, followed by HT with 22%, then DNN with 16%. The RFR model reports a 91% fault detection accuracy for the long duration, followed by HT with 24%, then DNN 16%. These results suggest that the RFR model is an appropriate model for detecting short, medium, and long fault durations. DNN requires a larger dataset to achieve optimal performance, which may explain its poor performance. We split the dataset into three parts with specific fault durations: 1. A short fault duration with 16,211 instances; 2. Medium fault duration with 21,500 instances; and 3. Long fault duration with 15,800 instances. Training and testing DNN on each sub-dataset was not sufficient for it to achieve optimal detection accuracy, suggesting that RFR can achieve its highest accuracy with a relatively small number of instances compared to the DNN model.

### 3.5. Experiment Results #3: Handling Missing Data

**Figure 7:**
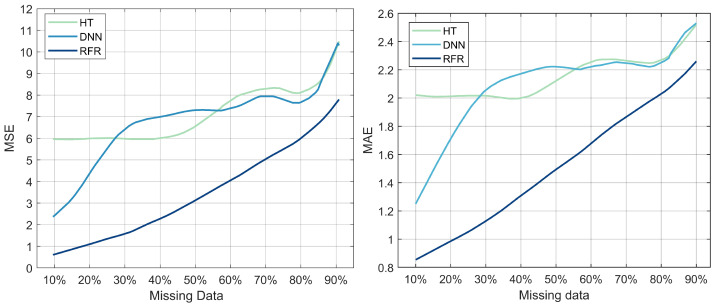
MSE and MAE as a function of the percentage of missing data for the three models: DNN, HT, and RFR.

Figure 7 illustrates MSE and MAE as a function of the percentage of missing data for the three selected models: DNN, RFR, and HT. The purpose of this experiment was to evaluate the model’s robustness when handling missing data. The collected measurements within a real power system network, including voltage, magnitude, and frequency, can be incomplete due to equipment failure, data storage issues, or unreliable communication [31]; therefore, it is crucial to evaluate the model’s capacity for accurately predicting the fault duration. The MSE of the three models increases as the percentage of missing data increases (Figure 7). RFR’s MSE values of 1.8 and 7.8 correlate to missing data percentages of 10% and 90%, respectively. DNN reports an MSE value of 2.5 with 10% of the data missing, while HT reports an MSE value of 6 for the same percentage of data missing. The MSE values of DNN and HT increase as the percentage of missing data increases, reaching the highest value of 10. Figure 7 also illustrates the MAE as a function of the percentage of missing data. The evolution of the MAE value for the three models indicates similar behavior to the previous one. RFR has an MAE value of 0.85, followed by DNN with 1.29, then HT with 2.1, with 10% of the data missing. The MAE values of the three models increase as the percentage of missing data increases to reach their highest values, which are 2.25 for RFR, 2.58 for HT, and DNN, with 90% of the data missing. These results suggest that the RFR model is more resilient and tolerant to missing data; therefore, it is the optimal model for fault duration prediction even with incomplete data.

### 3.6. Experiment Results #4: Handling Streaming Data

**Figure 8:**
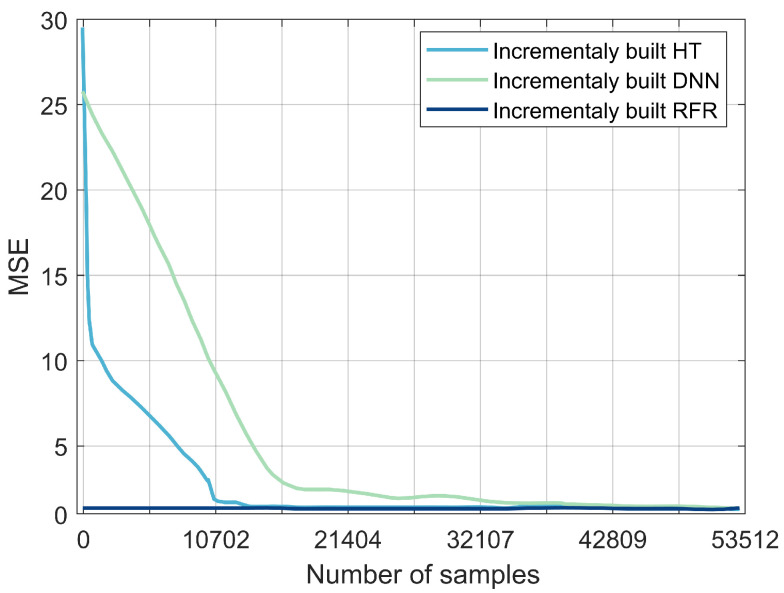
Comparison between DNN, HT, and RFR in terms of MSE.

The RFR, DNN, and HT models selected from the previous experiments were evaluated with streaming data. The models were trained incrementally: they were not trained and tested on the entire dataset, they were incrementally trained with one sample at a time. The MSE and the processing time of each model were then evaluated (Figure 8). The MSE of the RFR values were consistently below 0.1 s as the number of samples increased. For HT, the MSE dropped sharply from 28 s to 0.5 s; for samples between 0 and 10,702, it stagnated at 0.5 s, and then dropped to 0.1 s. The DNN’s MSE values decreased from 30 s to 2 s as the number of samples increased to 32,107, then decreased slowly to reach the lowest value of 0.1 s before stabilizing. RFR reported the lowest value for processing time per sample: 0.0028 ms, followed by DNN with 0.0032 ms, then HT with 0.7 ms. The results obtained in this experiment set suggest that RFR is a potential model for detecting fault locations within a near-real-time streaming environment. A summary of the obtained results for the four experiments is provided in Table 4. The overall accuracy, MAE, MSE, processing time, and overall rankings are high for RFR, medium for DNN, and low for the other models.

## 4. Discussion

Experimental results show that the performance of the ensemble method used in this paper, i.e., RFR, consistently outperforms the other models in simultaneously detecting the location and duration of faults on a multi-bus system. With an overall accuracy over 84%, the RFR model performed optimally and consistently at various fault locations as well as with various fault duration (short, medium, and long), suggesting that RFR is the appropriate model for this dual-purpose task. For the same task, DNN also demonstrated consistent overall performance, albeit at the expense of a long processing time that makes it unsuitable for real-time applications.

While machine learning models can perform fairly well in an ideal and deterministic environment that is free from anomalies, it was pragmatic and necessary to evaluate the performances of these models in scenarios where data might be missing due to equipment failure, data storage issues, or unreliable communication. Based on the results in this paper, all three models, i.e., HT, DNN, and RFR, show MSE and MAE values of under 11 and 2.6, respectively, when the percentage of missing data is progressively increased from 10% to 90%. Depending on the severity of the said factors, RFR was proven to be the optimal candidate, followed by HT and DNN, in extrapolating system status during non-steady-state operations.

Another critical component of a model’s capability, when deployed in a real-world environment, is its ability to evolve and adapt to unexpected data distribution changes and concept drifts. From the experimental results, RFR achieved an MSE 0.0028 ms when trained and tested incrementally on streaming data, which makes it suitable for detecting faults in near-real time. Overall, the RFR model performed optimally and consistently under four different scenarios, indicating that the model can generalize and adapt to new, previously unseen, data without the risk of overfitting or underfitting.

## 5. Conclusions

A random forest regression (RFR)-based model was successfully implemented to identify the location and duration of faults. Various fault scenarios were modeled using PNNL’s GridPACK software for the generation of the training dataset. A total of nine fault scenarios was simulated by injecting faults on specific buses over a specified period of time. The RFR models were trained and evaluated within the context of four study cases: detecting fault location, predicting fault duration, handling missing data, and streaming data. A comparison was also conducted between the RFR model and several state-of-the-art models using multiple performance metrics, including accuracy, MSE, and processing time. Results indicate that both RFR and DNN models are capable of detecting the location and duration of a fault with an accuracy of 84% and 72%, respectively. The RFR, DNN, and HT models yielded better results when predicting faults in streaming networks. Overall, the RFR model consistently outperformed the other models, making it appropriate for real-time situational awareness deployments to determine both the location and duration of the faults while handling missing data. Further work will be devoted to assessing the model’s scalability with respect to large bus systems as well as further improving its accuracy.

## Figures and Tables

**Table 1:** Existing literature on fault location and duration in power system.

Category	Approach	Fault Types	Advantages	Limitations
Conventional	Impedance-based [8,11]	Physical	Ease of implementation.	The accuracy can be affected in the case of a grounded fault where the fault resistance is high. The fault duration was not considered.
	Time wave-based [10]	Physical	Large resistance, load variance, grounding resistance, reflection, and refraction of the traveling wave and series capacitor bank.	The accuracy depends on the correctness of the line parameters’ estimated values, including capacitance and line inductance. The fault duration was not considered.
Machine learning	NN + Levenberg–Marquardt [12,13]	Physical	The detection error is less than 3%. High tolerance to the fault resistance, fault type, fault location, and the embedded remote-end source.	The convergence time for the training process is high. The fault duration was not considered.
	NN-based [14]	Physical	Optimal results in terms of estimating the fault distance from the sub-stations even under network–topological changes. High tolerance to noise.	Inappropriate for detecting fault location in a streaming power system network.
	CNN-based [15]	Physical	Optimal localization estimation even under low visibility (7% of buses).	The fault duration was not considered.
	RF + DT [16]	Physical	Fault location detection accuracy is 91% with a minimum number of buses (5–7%).	
	RF [17]	Physical	Fault location detection accuracy is 90.96% in distribution systems.	
	MLE + DBSCAN [19]	Physical	The proposed data cleansing approach outperforms Chevyshev and K means and achieve a precision of 95%. Less than 0.9 s to classify event for a typical window size of 30 sample data.	
	KNN [18]	Physical	Fault location accuracy reaches 98.70% with an error between 0.61% and 6.5%.	The proposed model was trained/tested on the PV system only.
	HAT + DDM + ADWIN [29,30]	Physical and cyber	Classification accuracy is greater than 94% for multiclass and greater than 98% for binary class. Adaptable to the concept of drift events.	The fault location and duration were not considered.
	RNN-based [5], NBC + SVM [6]	Physical	Predicting fault duration with 97% accuracy. The RNN-based approach is suitable for a real-time environment.	The fault location was not considered.
Hybrid	Wavelet transform + SVM [20]	Physical	The fault classification error is below 1% for all fault types. The overall error is 0.26% for SLG, 0.74% for LLG, 0.20% for LL, and 0.39% for LLLG.	The fault duration was not considered. Not suitable for streaming power system data. The accuracy of the SVM depends on selecting and tuning the appropriate kernel type and hyper-parameters.
	Wavelet analysis + K-means +ELE [21]	Physical	Fault location accuracy attained 100%.	The fault duration was not considered.
	Wavelet analysis + Fuzzy logic [22]	Physical	The error between the actual fault location and the predicted one is low than 0.002%.	
	Discrete wavelet transform+ SVM [23]	Physical	Fault location accuracy is 98.27% for IEEE 13-bus and 98.29% for the IEEE 34-bus test systems.	

**Table 2:** Common three-phase fault modeling for nine scenarios with different duration.

Scenario	Fault Location	Fault Duration	Simulation Time	Number of Generated Sample for Each Fault Duration	Number of Generated Samples for Each Scenario
Scenario 1–9	Apply fault at bus 1–9	0.05 s to 0.5 s with a step of 0.05 s	10 s	594 samples	5945 samples/scenario. Total number of samples is 53,512

**Table 3:** Hypertuning parameters for KNN, RF, DNN, DT, NB, HT, NN, and SVM.

Model	Hyperparameters			Mean Squared Error	Standard Deviation	Model	Hyperparameters			Mean Squared Error	Standard Deviation
KNN	**Weight** **function**	Uniform	1	11.21	2.6	RFR	Max feature: sqrt	Number of trees	1	10.31	2.68
			10	7.25	0.45				10	6.45	0.53
			100	6.71	0.17				100	6.2	0.67
		**Distance**	1	11.21	2.6		**Max feature:** **log2**	**Number of** **trees**	1	10.52	2.68
			10	7.24	0.43				10	6.75	1.26
			**100**	**6.7**	**0.16**				**100**	**6.15**	**0.63**
SVM	Polynomial kernel	C=1		6.013	0.11	NN	**Relu** **function**	**Number of** **hidden nodes**	**150**	**4.37**	**0.18**
		C=5		6.13	0.14				300	4.64	0.23
		C=10		6.16	0.08				450	4.62	0.12
	**Radial** **basis function ** **(RBF) kernel**	C=1		6.09	0.14		Identity function	Number of hidden nodes	150	6.15	0.08
		C=5		6.17	0.08				300	6.15	0.06
		**C=10**		**5.9**	**0.1**				450	6.16	0.08
DT	Minimum leaf size = 1	Random state	0	10.51	3.56	NB	**Alpha = 1 × 10^−6^**	**Lambda**	1 × 10^−6^	1.26 × 10^−3^	1.42 × 10^−4^
			1	10.39	3.65				1 × 10^−4^	1.17 × 10^−3^	1.56 × 10^−4^
			2	10.58	3.57				**1** × 10^−2^	**1.07** × 10^−3^	**1.66** × 10^−4^
	**Minimum leaf** **size = 6**	**Random state**	0	9.32	3.15		Alpha = 1 × 10^−4^	Lambda	1 × 10^−6^	1.14 × 10^−3^	1.98 × 10^−4^
			**1**	**9.29**	**3.12**				1 × 10^−4^	1.19 × 10^−3^	1.51 × 10^−4^
			2	9.31	3.15				1 × 10^−2^	1.15 × 10^−3^	2.37 × 10^−4^
DNN	**Relu function**	**5 hidden layers**	50 hidden nodes	1.20 × 10^−2^	2.40 × 10^−3^	HT	Split function: Gini Index	Split confidence	1 × 10^−5^	12.41	4.88
			**100 hidden** **nodes**	**1.12** × 10^−2^	**1.39** × 10^−3^				1 × 10^−4^	14.53	6.13
			150 hidden nodes	1.14 × 10^−2^	1.39 × 10^−3^				1 × 10^−3^	14.91	6.24
		10 hidden layers	50 hidden nodes	1.12 × 10^−2^	3.51 × 10^−3^		**Split function:** **Information gain**	**Split** **confidence**	**1** × 10^−5^	**10.88**	**2.89**
			100 hidden nodes	1.14 × 10^−2^	1.39 × 10^−3^				1 × 10^−4^	11.24	8.13
			100 hidden nodes	1.20 × 10^−2^	2.40 × 10^−3^				1 × 10^−3^	17.64	7.22

**Table 4:** Summary of the RFR’s performances compared to those of DNN, HT, NN, SVM, DT, NB, and KNN, obtained in the four experiments.

Experiment	Performance Metrics	RFR	DNN	HT	NN	SVM	DT	NB	KNN
1. Detecting fault location	Overall accuracy for four fault locations	84%	72.5%	27%	18.75%	14%	2%	8.25%	41%
2. Predicting fault duration	MSE	1.1 s	1.2 s	1.1 s	5.6 s	6.5 s	6.6 s	6.2 s	5.1 s
	MAE	0.6 s	0.6 s	0.6 s	1.9 s	2.2 s	2.5 s	2.2 s	1.8 s
3. Handling missing data	MSE	4.6 s	8.4 s	8.7 s	-	-	-	-	-
	MAE	1.5 s	2.09 s	2.14 s	-	-	-	-	-
4. Detecting fault in streaming data	Processing time per sample	0.0028 ms	0.0032 ms	0.7 ms	-	-	-	-	-
Overall ranking		High	Medium	Low	Low	Low	Low	Low	Low

## Abbreviations

The following abbreviations are used in this manuscript:

AM	Active management
BPN	Back-propagation neural network
CNN	Convolutional neural network
DBSCAN	Density-based spatial clustering and application with noise
DLG	Double line to ground
DNN	Deep neural network
DT	Decision tree
DWT	Discrete wavelet transform
ELE	Event location estimation
EZ	Electrical zone
FNN	Feedforward neural network
GA	Genetic algorithm
GPS	Global Positioning System
HPC	High-performance computing
HT	Hoeffding tree
KNN	k-nearest neighbors
LL	Line to line
LLL	Three phase to ground
MAE	Mean absolute error
ML	Machine learning
MLE	Maximum likelihood estimation
MSE	Mean squared error
MTHVDC	Multi-terminal high-voltage direct current
MWE	Modified wavelet energy
NB	Naive Bayes
NBC	Naive Bayes classifier
PDT	Physics-based decision tree
PMU	Phasor measurement unit
PNN	Probabilistic neural network
PV	Photovolatic
RF	Random forest
RFR	Random forest regressor
RNN	Recurrent neural network
SE	Shannon’s entropy
SLG	Single line to ground
SVM	Support vector machine

## References

[1] Haes Alhelou H., Hamedani-Golshan M.E., Njenda T.C., Siano P. (2019). A survey on power system blackout and cascading events: Research motivations and challenges. Energies.

[2] Salimian M.R., Aghamohammadi M.R. (2017). A three stages decision tree-based intelligent blackout predictor for power systems using brittleness indices. IEEE Trans. Smart Grid.

[3] Zhang Y., Xu Y., Dong Z.Y. (2017). Robust ensemble data analytics for incomplete PMU measurements-based power system stability assessment. IEEE Trans. Power Syst..

[4] Lawton L., Sullivan M., Van Liere K., Katz A., Eto J. (2003). A Framework and Review of Customer Outage Costs: Integration and Analysis of Electric Utility Outage Cost Surveys.

[5] Jaech A., Zhang B., Ostendorf M., Kirschen D.S. (2018). Real-time prediction of the duration of distribution system outages. IEEE Trans. Power Syst..

[6] Joyokusumo I., Putra H., Fatchurrahman R. A Machine Learning-Based Strategy For Predicting The Fault Recovery Duration Class In Electric Power Transmission System. Proceedings of the 2020 International Conference on Technology and Policy in Energy and Electric Power (ICT-PEP).

[7] Gururajapathy S.S., Mokhlis H., Illias H.A. (2017). Fault location and detection techniques in power distribution systems with distributed generation: A review. Renew. Sustain. Energy Rev..

[8] Ajenikoko G.A., Sangotola S.O. (2016). An overview of impedance-based fault location techniques in electrical power-transmission network. Int. J. Adv. Eng. Res. Appl. (IJA-ERA).

[9] Lim P.K., Dorr D.S. Understanding and resolving voltage sag related problems for sensitive industrial customers. Proceedings of the 2000 IEEE Power Engineering Society Winter Meeting. Conference Proceedings (Cat. No. 00CH37077).

[10] Ma G., Jiang L., Zhou K., Xu G. (2016). A Method of line fault location based on traveling wave theory. Int. J. Control Autom..

[11] Alwash S.F., Ramachandaramurthy V.K., Mithulananthan N. (2014). Fault-location scheme for power distribution system with distributed generation. IEEE Trans. Power Deliv..

[12] Javadian S.A.M., Nasrabadi A.M., Haghifam M.R., Rezvantalab J. Determining fault’s type and accurate location in distribution systems with DG using MLP Neural networks. Proceedings of the 2009 International Conference on Clean Electrical Power.

[13] Aslan Y. (2012). An alternative approach to fault location on power distribution feeders with embedded remote-end power generation using artificial neural networks. Electr. Eng..

[14] Dehghani F., Nezami H. A new fault location technique on radial distribution systems using artificial neural network. Proceedings of the 22nd International Conference and Exhibition on Electricity Distribution (CIRED 2013).

[15] Li W., Deka D., Chertkov M., Wang M. (2019). Real-Time Faulted Line Localization and PMU Placement in Power Systems Through Convolutional Neural Networks. IEEE Trans. Power Syst..

[16] Zainab A., Refaat S.S., Syed D., Ghrayeb A., Abu-Rub H. Faulted Line Identification and Localization in Power System using Machine Learning Techniques. Proceedings of the 2019 IEEE International Conference on Big Data (Big Data).

[17] Okumus H., Nuroglu F.M. (2021). A random forest-based approach for fault location detection in distribution systems. Electr. Eng..

[18] Madeti S.R., Singh S. (2018). Modeling of PV system based on experimental data for fault detection using kNN method. Sol. Energy.

[19] Pandey S., Srivastava A., Amidan B. (2020). A Real Time Event Detection, Classification and Localization using Synchrophasor Data. IEEE Trans. Power Syst..

[20] Ekici S. (2012). Support Vector Machines for classification and locating faults on transmission lines. Appl. Soft Comput..

[21] Kim D.I., White A., Shin Y.J. (2018). Pmu-based event localization technique for wide-area power system. IEEE Trans. Power Syst..

[22] Hossam-Eldin A., Lotfy A., Elgamal M., Ebeed M. Combined traveling wave and fuzzy logic based fault location in multi-terminal HVDC systems. Proceedings of the 2016 IEEE 16th International Conference on Environment and Electrical Engineering (EEEIC).

[23] Mohammadnian Y., Amraee T., Soroudi A. (2019). Fault detection in distribution networks in presence of distributed generations using a data mining–driven wavelet transform. IET Smart Grid.

[24] Chow M.Y., Taylor L.S., Chow M.S. (1996). Time of outage restoration analysis in distribution systems. IEEE Trans. Power Deliv..

[25] Palmer B., Perkins W., Chen Y., Jin S., Callahan D., Glass K., Diao R., Rice M., Elbert S., Vallem M. (2016). GridPACKTM: A framework for developing power grid simulations on high-performance computing platforms. Int. J. High Perform. Comput. Appl..

[26] Muallem A., Shetty S., Pan J.W., Zhao J., Biswal B. (2017). Hoeffding Tree Algorithms for Anomaly Detection in Streaming Datasets: A Survey. J. Inf. Secur..

[27] He Y., Mendis G.J., Wei J. (2017). Real-Time Detection of False Data Injection Attacks in Smart Grid: A Deep Learning-Based Intelligent Mechanism. IEEE Trans. Smart Grid.

[28] Mrabet Z.E., Selvaraj D.F., Ranganathan P. Adaptive Hoeffding Tree with Transfer Learning for Streaming Synchrophasor Data Sets. Proceedings of the 2019 IEEE International Conference on Big Data (Big Data).

[29] Dahal N., Abuomar O., King R., Madani V. (2015). Event stream processing for improved situational awareness in the smart grid. Expert Syst. Appl..

[30] Adhikari U., Morris T.H., Pan S. (2017). Applying hoeffding adaptive trees for real-time cyber-power event and intrusion classification. IEEE Trans. Smart Grid.

[31] Breiman L. (2001). Random Forests. Mach. Learn..

[32] Glocker B., Pauly O., Konukoglu E., Criminisi A. (2012). Joint classification-regression forests for spatially structured multi-object segmentation. Proceedings of the European Conference on Computer Vision.

[33] Linusson H. (2013). Multi-Output Random Forests.

[34] Pauly O. (2012). Random Forests for Medical Applications. Ph.D. Thesis.

[35] Paper D., Paper D. (2020). Scikit-Learn Regression Tuning. Hands-On Scikit-Learn for Machine Learning Applications: Data Science Fundamentals with Python.

[36] Cheng R., Fang Y., Renz M. (2014). Data Classification: Algorithms and Applications.

